# Reconciling research and implementation in micro health insurance experiments in India: study protocol for a randomized controlled trial

**DOI:** 10.1186/1745-6215-12-224

**Published:** 2011-10-11

**Authors:** Conor Doyle, Pradeep Panda, Ellen Van de Poel, Ralf Radermacher, David M Dror

**Affiliations:** 1 Micro Insurance Academy, 246 Sant Nagar, East of Kailash, New Delhi 110065, India; 2 Institute of Health Policy and Management, Erasmus University Rotterdam, P.O. Box 1738, 3000 DR Rotterdam, The Netherlands

**Keywords:** Micro insurance, community based, healthcare utilisation, financial protection, randomised trial

## Abstract

**Background:**

Microinsurance or Community-Based Health Insurance is a promising healthcare financing mechanism, which is increasingly applied to aid rural poor persons in low-income countries. Robust empirical evidence on the causal relations between Community-Based Health Insurance and healthcare utilisation, financial protection and other areas is scarce and necessary. This paper contains a discussion of the research design of three Cluster Randomised Controlled Trials in India to measure the impact of Community-Based Health Insurance on several outcomes.

**Methods/Design:**

Each trial sets up a Community-Based Health Insurance scheme among a group of micro-finance affiliate families. Villages are grouped into clusters which are congruous with pre-existing social groupings. These clusters are randomly assigned to one of three waves of implementation, ensuring the entire population is offered Community-Based Health Insurance by the end of the experiment. Each wave of treatment is preceded by a round of mixed methods evaluation, with quantitative, qualitative and spatial evidence on impact collected. Improving upon practices in published Cluster Randomised Controlled Trial literature, we detail how research design decisions have ensured that both the households offered insurance and the implementers of the Community-Based Health Insurance scheme operate in an environment replicating a non-experimental implementation.

**Discussion:**

When a Cluster Randomised Controlled Trial involves randomizing within a community, generating adequate and valid conclusions requires that the research design must be made congruous with social structures within the target population, to ensure that such trials are conducted in an implementing environment which is a suitable analogue to that of a non-experimental implementing environment.

## Background and Rationale

### Background

Health insurance coverage is woefully lacking in the developing world. Poor households must often resort to high-cost loans or asset sales to finance healthcare, and may be forced to forego essential treatments altogether (Binnedijk E, *et al*, Hardship financing of healthcare among rural poor in Orissa, India, submitted). Development organisations have increasingly recognised the role that health microinsurance (HMI) can play as a poverty reduction tool [1,2]. One form of HMI is Community-Based Health Insurance (CBHI), in which communities mutualise risks and resources into a locally-managed healthcare fund [3]. CBHI schemes have been implemented in India, Afghanistan, Nepal, Burkina Faso, Mali, Senegal, Nigeria, and extensively throughout Rwanda and Tanzania. However, recent literature reviews have noted both a limited body of evidence on both HMI and CBHI impact, and considerable methodological problems in many of the available studies [4-6].

To help close knowledge gaps and aid policy design, Erasmus University Rotterdam, the University of Cologne and the Micro Insurance Academy (MIA) are operating three separate CBHI impact evaluations in rural areas of northern India. The microinsurance schemes are being implemented by three Indian charitable NGOs (BAIF, Nidan and Shramik Bharti) with technical support from MIA. Each evaluation is organised as a cluster randomised controlled trial (CRCT), in which randomly selected members of a network of women's microfinance groups ("Self Help Groups" or SHGs) are offered the option to affiliate to a CBHI scheme which they design and manage. The impact of each scheme on a range of indicators will be analysed, including healthcare utilisation and financial protection of members.

This article describes the study protocol of these trials, with a focus on the issues faced in designing a methodologically robust CRCT that must also meet the constraints imposed by implementation requirements. Scientific evaluations of development programs must always take place in a specific context, with the result that the institutional setting and implementation needs of the program can influence both the internal and external validity of analyses of its impact. The article details how implementation constraints impacted research design, and how research and implementation needs were jointly considered prior to launching the project, in order to ensure both a supportive and scientifically robust trial environment.

### Rationale

The crucial issue in measuring the impact of CBHI is the construction of a comparable and unbiased counter-factual. Let Y_i _be annual health care use in a village i that is offered the opportunity to enroll in a CBHI scheme. The village has two potential outcomes; its annual health care use if it avails of CBHI (treated, or y_it_) and its annual health care use if it does not (control, or y_ic_). The treatment effect of CBHI on annual village level health care use equals y_it_-y_ic_; but only one of the potential outcomes can be observed, as a single village cannot simultaneously be insured and uninsured. If multiple villages are observed, some which avail of CBHI and others which do not, an estimate of the expected treatment effect, E(y_it_-y_ic_), can be recovered by measuring the difference between them, (Y_t_-Y_c_). For this to provide unbiased estimates, the villages that take up CBHI must be comparable, both in observable and unobservable characteristics, to those which do not. However, in the context of a voluntary insurance program, those villages which take up CBHI are likely to be richer, sicker, or otherwise systematically different from those which do not. The effects of these selection biases over treatment and control groups, B, means the measured effect of CBHI is (Y_t_-Y_c_) = E(y_it_-y_ic_)+B.

One way to avoid this problem is by randomising villages into treatment and control groups. Cluster randomised controlled trials (also known as "Group Randomised Trials"), ensure an unbiased counterfactual by explicitly randomising treatment over clusters of individuals. In the above context, the treatment is the offer of CBHI, and the groups/clusters are villages. As external variables are blocked from influencing selection into treatment and control groups, the outcomes of villages randomly assigned to the control group are the same in expectation as the outcomes the treatment group would have experienced had they remained untreated i.e. E(B) = 0. The measured treatment effect, (Y_t_-Y_c_), is thus an unbiased estimate of the mean treatment effect, E(y_it_-y_ic_) [7,8]*. For this reason, randomised trials are generally considered the "gold standard" in quantitative impact evaluations [9].

Unfortunately, by this standard, the quantitative knowledge base on the impact of CBHI is methodologically weak, as well as limited in scope. We are aware of only 6 CBHI schemes which have been subject to quantitative evaluations employing regressions or more rigorous methods. Only 1 scheme has been evaluated by CRCT [10], with 2 other schemes having been evaluated using a less rigorous quasi-experimental approach [11]. The remaining 3 schemes have been evaluated only via regression-based methodologies [12-14], which cannot control for unobservable differences between insured and uninsured populations, and are therefore likely to deliver biased impact estimates. The body of available evidence on CBHI impact is too small and methodologically weak to make any general conclusions. Moreover, these six evaluations each utilise different indicators to measure the impact of CBHI, which causes considerable difficulties in making cross-study comparisons of impact.

The motivation for the three CRCTs described in this paper is to help close these knowledge gaps: to provide reliable quantitative evidence on CBHI impact by employing the CRCT approach; to quickly upscale the number of CBHI trials which have been evaluated by running three separate trials; and to begin generating evidence which is comparable across trials by using standard data collection tools and outcome indicators.

## Methods

All three CRCTs follow the same research protocol. Pre-defined clusters of individuals are randomly assigned to either treatment or control groups. Those in the treatment clusters take part in a structured education program on insurance and CBHI, participate in package design and pricing decisions, and are then offered the option to pay a premium and join the CBHI scheme. Those paying the premium enjoy pre-defined health insurance benefits for one year. Those in control clusters are offered neither insurance education nor CBHI membership. Three waves of implementation sequentially roll out treatment to all clusters over time. The following discussion focuses on how the research protocol has been pro-actively developed to take into account implementation considerations.

### Location, Implementing Partners & Target Population

**Figure 1:**
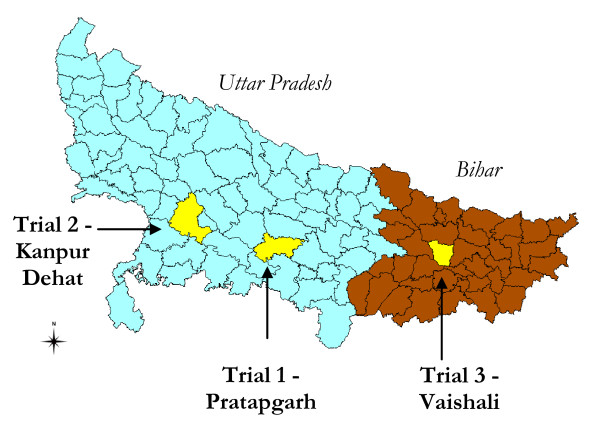
**Locations of Trial Sites**.

The three CRCTs are being undertaken at three separate sites in Northern India - one each in Kanpur Dehat and Pratapgarh districts in Uttar Pradesh, and one in Vaishali district in Bihar (Figure 1). All three sites are poor rural areas primarily dependent on agriculture, located 50-100 km outside major urban centers.

Implementation of a CBHI scheme pre-supposes that the trial region contains a self-identified community, with experience of co-operating democratically for mutual benefit, and an apex body within this community capable of driving new social programs. At each trial, implementation is thus facilitated by a locally based NGO, or "implementing partner". Each implementing partner oversees a pre-existing network of Self Help Groups (SHGs) in the project area. SHGs typically consist of a group of 10-20 women from a village, who agree to add a pre-specified amount of money to a communal pot/bank account each month. Members may take loans from the pot for investment activities and emergency expenditures. The SHG concept originated in India in the 1980s as a self-contained alternative to government sponsored co-operative societies [15]. The number of SHGs has grown steadily since the early 1990's, and groups are now found throughout India [16]. In 2009, over 1.6 million functioning SHGs were affiliated with formal-sector banks [17]. Implementing partners for our CBHI project were purposively selected on the basis of an evaluation of their capacity to provide support to their SHGs during the insurance set-up process^† ^.

At each site, the experimental cohort is defined as all members of households with at least one woman registered in March 2010 as a member of an SHG facilitated by the local implementing partner. Totals of 8933, 7105 and 7838 persons are eligible to take part in the experiment at Pratapgarh, Kanpur Dehat and Vaishali respectively. Table 1 presents summary statistics of those eligible to participate in the trial (based on data from the baseline survey - see below). The average daily consumption per person (in purchasing power parity $, including home produced food) is estimated at $2.72 (Pratapgarh), $3.05 (Vaishali) and $4.17 (Kanpur Dehat). Respectively, 26%, 18% and 23% of households report themselves as being below the international absolute poverty line of PPP $1.25 per person per day. The majority of adult males work in agriculture, either farming their own small holdings or working as casual labourers on others' land. At Pratapgarh and Vaishali, which include some small rural town areas, 19%-20% of adult males work in a small business, such as roadside vending. In Kanpur Dehat, 70% of all adult males work in agriculture, and only 12% work in any form of business. At all sites, most adult females attend to domestic duties for their households. Healthcare access is poor: as is common in India, when a new illness develops, at least half of first-contact outpatient visits at all three sites are to unqualified providers [18].

**Table 1:** Summary Statistics of Families of SHG Members by Trial Sites

	Trial 1 - Pratapgarh	Trial 2 - Kanpur Dehat	Trial 3 - Vaishali
State	Uttar Pradesh	Uttar Pradesh	Bihar

Implementing Partner	BAIF	Shramik Bharti	Nidan

No. Villages	15	42	34

Trial Population*	8933	7105	7838

No. SHG Members	1557	1226	1459

Mean Annual Consumption per capita Rs (*Standard Deviation*)	16233 (15131)	24934 (30298)	18231 (21772)

Mean Annual Consumption per capita $US (*Standard Deviation*)**	358 *(334)*	551 *(669)*	403 *(481)*

Mean Consumption, per capita per annum, $PPP (*Standard Deviation*)***	991 *(924)*	1523 *(1850)*	1113 *(1329)*

Mean Daily Consumption per capita, $PPP (*Standard Deviation*)	2.72 (*2.53*)	4.17 (*5.07*)	3.05 (*3.64*)

% of HHs below International Absolute Poverty Line (PPP "tabcaption".25 at 2005 prices)	26	18	23

% Adult Males in Agriculture	53	70	59

% Adult Females at Home	65	70	67

% First Visits to Unqualified Providers	55	67	50

* Estimate - Number of SHG members is known, but not all SHG members answered a HH survey, so size of all households is not known. Estimate = [(No. of SHG members who did not answer HH survey) × (average HH size at site)] + [No. people in HHs of SHG members who answered HH survey]

** Calculated at USD/Rupee midrate of 1/45.29, Dec 3 2010

*** Calculated at World Bank PPP conversion factor for 2009 of 0.3616

### Description of treatment program

**Figure 2:**
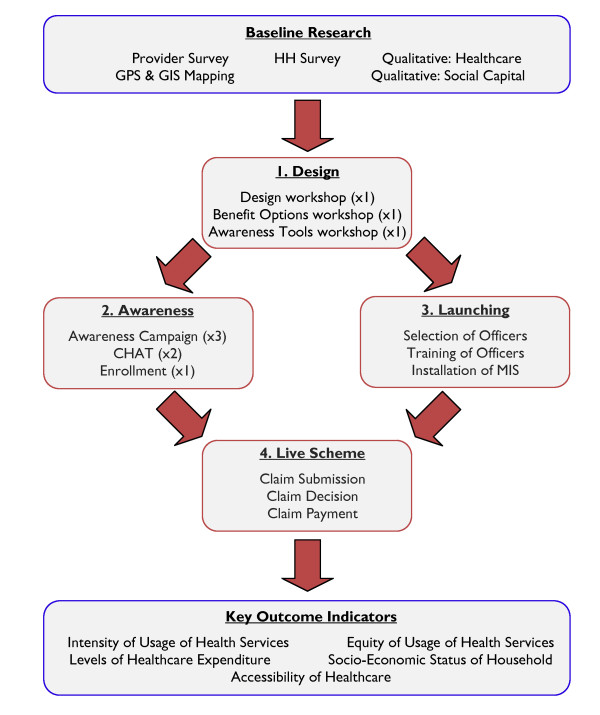
**CBHI Implementation & Research Processes**.

The treatment on these CRCTs is a CBHI implementation program which has been developed by the Micro Insurance Academy. The MIA is an Indian-based charitable trust which provides research, training, structured technical assistance, and advisory services to CBHI and other microinsurance programs. In a CBHI scheme, a local community owns and operates a health insurance fund on a not-for-profit basis. Community members are involved in package design and pricing decisions, and ideally elect/nominate members to act as administrative staff. Any profits are retained as capital buffers, or returned to members. The particular CBHI implementation model used is based on the MIA's extensive field experience in the set-up of CBHI schemes, and has previously been rolled-out in full at four sites, two in India and two in Nepal. Although it has proven successful in establishing CBHI schemes among low income persons with little or no prior experience of insurance, this model's impact on access to care and financial protection has not previously been the subject of rigorous evaluation. The model consists of a sequence of four self-contained modules (Figure 2). To aid generalisability of findings, and comparisons across the three trials, each module is defined by function (the purpose it seeks to achieve) rather than content (the specific manner in which this purpose is achieved) [19]. The sequencing and time span of each module is homogeneous across sites, but content and operations are subject to (usually minor) context specific modifications.

Implementation begins with a "Design" module, which optimises the contents and operation of the CBHI scheme, aiming to secure community buy-in and scheme sustainability. SHG leaders and scheme staff from the implementing partner are taken through 3 structured workshops. First, a Design workshop develops business processes for operational aspects of CBHI (premium collection, claims processing, etc.), integrating them into the existing NGO and SHG management structures. Second, a Benefit Options Finalisation workshop engages SHG leaders to design up to 5 potential CBHI coverage packages. A simple MS-Excel interface allows participants to cost a profit-neutral premium for any combination of coverage options and caps, reflecting the prices and illness incidences reported in a baseline survey (see below). Third, the same audience is exposed to a range of pilot insurance education tools (posters, songs, street plays, videos and a movie) at an Awareness Tools workshop, and feedback gathered.

The "Awareness" module is then rolled out to the treatment clusters. This module structures enrolment decisions via a program of 6 fortnightly SHG meetings. The first 3 discussions ("awareness campaign") are structured educational sessions explaining the concept and workings of insurance, intended for SHG members and the financial decision makers in their households. Two further structured discussions introduce households to the available CBHI packages and ask each SHG to cast a vote for one preferred package ("Choosing Healthplans All Together" or CHAT). SHG votes are aggregated to choose one insurance package for the trial. In one further group meeting, the price and coverage of the selected package are explained. Treatment group members can enroll in CBHI at this meeting, or for a limited period afterwards. Enrolment decisions are intended to be made en-bloc: the members of each SHG must decide either to join the insurance scheme as a group, along with all the members of their households, or not enroll at all. En-bloc enrollment helps to avoid adverse selection. Enrollment decisions are voluntary, but necessarily public, as SHG members must debate among themselves whether to join.

The simultaneous "Launching" module sets up back-office and operations elements of the scheme. Processes are formalised, and forms developed. Local variations in specific processes are allowed. Five levels of staff are elected from among SHG members and implementing partner staff (local insurance activists, insurance coordinator, claims committee, coordination committee, and ombudsman). Multi-day trainings are held on-site for each cadre of staff. A scheme office is set up at a focal point within the project area, facilitated with phones and computers. A dedicated microinsurance management information system (MIS) application is delivered by MIA to the implementing partner, and separate trainings on its use are held.

Finally, once Awareness and Launching modules are complete, the scheme goes live. Members are supplied with pre-printed claim forms and printed materials describing benefits. Insurance activists, present in all clusters, assist CBHI members in submitting claims and receiving payouts. All claims are transmitted to the Claims Committee, composed of locally-elected leaders, who meet monthly to decide on payouts. Successful claims are paid in cash to the claimants. The separately elected ombudsman mediates any disputes which arise over claims decisions.

### Objectives & outcome measures

The key objective of these trials is to examine the effect of CBHI on members' usage of healthcare services and healthcare financing patterns. We put forward two primary hypotheses:

1. Members of a CBHI scheme will increase their utilization of available healthcare, as compared to those without insurance cover, for those categories of treatment which are covered by the insurance package.

2. Membership in a CBHI scheme is associated with a decrease in the household's Out-of-Pocket Spending (OOPS) on healthcare, as compared to those without insurance cover.

Two analogous primary outcome measures are used:

1. Number of consultations with a physician per case of illness or injury

2. Net OOPS per case of illness or injury = Expenditure on (Fees + cost of prescribed drugs + cost of recommended tests + cost of other recommended materials/procedures + insurance premium). OOPS does not include, transport, accommodation, other indirect costs of attaining healthcare, self-treatment, costs reimbursed by insurance

A variety of additional measures will be used to help assess healthcare usage in terms of the range of services utilised by those seeking care. Beyond these areas of core study, a rich set of quantitative, qualitative and spatial data which is being collected will enable researchers to examine secondary questions of the effect of CBHI on households' risk exposure, healthcare financing methods, probability of catastrophic health expenditures, consumption and investment patterns, and physical accessibility of healthcare.

### Method of Clustering

The first step in experimental design was to define the units which would be used for randomisation. Building from the smallest unit upwards, individuals, households, SHGs and villages are not suitable: the first three provide inaccurate analogues of the implementation environment which would prevail in a non-experimental implementation; the fourth additionally causes research issues. Individuals and households are unsuitable units of randomisation because the treatment program is designed to be administered to and managed by groups of geographically proximate people with previous associations to each other. The SHG is an unsuitable unit because villages typically contain multiple SHGs, and members of control group SHGs with a strong preference for insurance may push treatment group members to include them. This may be difficult to resist within the social structure of a small village. Implementing partners further advised that randomisation at a sub-village level would be perceived as capricious by the target population, and would damage uptake rates. Villages are an unsuitable unit of randomisation because SHG membership levels are highly unbalanced over villages (Table 2): the largest villages contain up to 18 times as many members as the smallest. Randomising by village would open the possibility of very small treatment groups in the first wave, creating problems of statistical power, possibly weakening implementation structures, and leaving the insurance scheme more financially exposed to insolvency.

**Table 2:** Summary Research Statistics by Trial Sites

	Trial 1 - Pratapgarh	Trial 2 - Kanpur Dehat	Trial 3 - Vaishali
No. SHG Members	1557	1226	1459

No. Households^#^	1455	1156	1454

No HH surveys complete	1284	1042	1363

No. Individuals^##^	8933	7105	7838

Smallest village: Largest village	1:17	1:8	1:18

No. Villages	15	42	34

No. Clusters	15	17	16

Average HHs per cluster	86	61	84

Minimum Detectable Effect Size	0.406	0.397	0.394

# Estimate - As a HH may contain more than one SHG member, we do not know how many HHs are among the SHG members who did not answer surveys. Estimate = [Counted number of households]+[(No of SHG members not answering a survey)*(1-proportion of households containing more than 1 SHG member among HH survey respondents)]

## Estimate - Number of SHG members is known, but not all SHG members answered a HH survey, so size of all households is not known. Estimate = [(No. of SHG members who did not answer HH survey) × (average HH size at site)] + [No. people in HHs of SHG members who answered HH survey]

**Figure 3:**
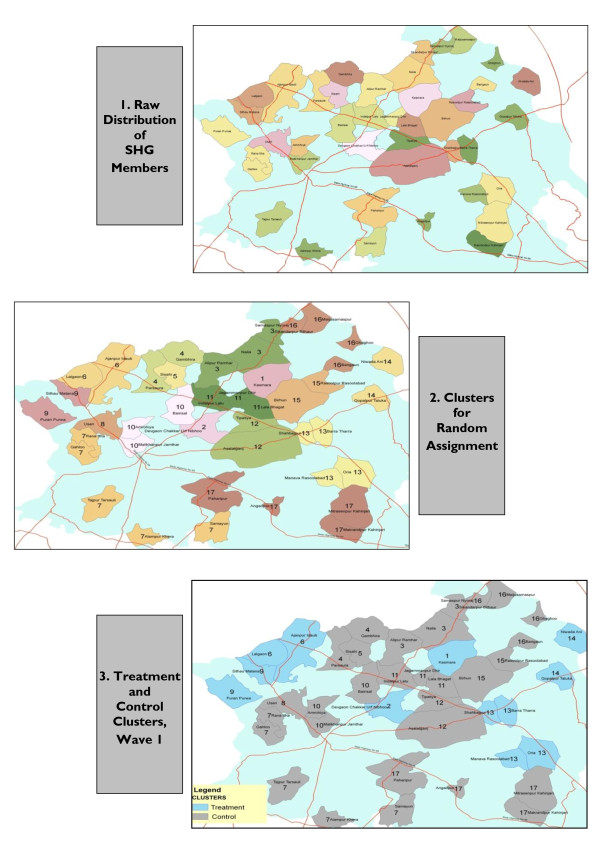
**Illustration of Clustering and Randomisation Process at Trial 2 (Kanpur Dehat)**. In panel 1, each coloured and bounded area is one village. In Panel 2, villages marked in the same colour and with the same number, form a cluster.

A group of villages is thus the smallest feasible cluster/unit of randomisation. Researchers developed a technique to cluster villages together which explicitly addresses the implementation constraints outlined above i.e. communities must not be subdivided, clusters must be geographically compact, and the insurance scheme must have a roughly equal sized pool of potential members whatever the outcome of randomisation. Baseline records of number of household surveys completed by SHG members were combined with GIS maps of the implementation area to show the spatial distribution of the trial population over villages (see Figure 3, panel 1 for the map used at Kanpur Dehat) ‡. Three criteria were then used to group villages into clusters:

**• Non-Divisibility: **A village cannot be divided over different clusters.

**• Equal Size: **Clusters must contain (roughly) equal numbers of SHG members. In interaction with the non-divisibility criterion, this defines a target cluster size, X:

X≈HighestNo.CompleteHHsurveysataSingleVillage

Note that the cluster size is defined as number of baseline household surveys completed by SHG members (rather than number of SHG members) as this is the effective number of household-level observations per cluster.

**• Continuity: **Each cluster should be geographically continuous. Where this is not possible, a cluster must be formed from proximate villages, such that no external village lies on a straight line between any two villages in the cluster.

15, 17 and 16 clusters were defined at Pratapgarh, Kanpur Dehat and Vaishali respectively (see Figure 3, panel 2 for the configuration determined at Kanpur Dehat). Clusters contain 60-80 households each (Table 2).

### Power calculations

Sample size on these trials is pre-determined by the number of persons meeting the inclusion criteria i.e. the number of persons in households in which at least one member has chosen to affiliate to an SHG. Researchers have designed the trials to maximize their ability to detect outcomes within the proscribed sample size. Power calculations can provide a useful indication of the probability that, given a certain study design, researchers will be able to reject the hypothesis of zero effect when an effect in fact exists. Using a priori information on mean and variability of the outcomes of interest and the correlation in these outcomes between households in the same clusters, one can calculate, for a given study design, the minimum detectable effect size (MDES) of the study. The standardised MDES for an outcome of interest, *y*, given a study design that has *J *clusters of identical size *n*, and a proportion *P *of the sample that is considered treated, can be calculated as [7]:

$$MDES_{y}=\frac{t_{\alpha ∕2}+t_{1-\kappa }}{\sqrt{P\left(1-P\right)J}}\sqrt{\rho +\frac{1-\rho }{n}}$$

where *ρ *is the correlation in *y *between households in the same cluster, *κ *represents the probability that we reject the null hypothesis of no effect when it is in fact false (power), *α *is the significance level and t_α/2 _and t_1-κ _are given by t-tables. This equation illustrates that the MDES is very dependent on the number of clusters (*J*), while cluster size (*n*) affects MDES much less; especially when households within clusters are quite similar (*ρ *is large). In sum, when individuals in clusters are very alike, having more clusters is preferred to having more people within clusters [20]. However, creating clusters smaller than groups of villages complicates the implementation of the CBHI scheme. Therefore several negotiation rounds have taken place between implementing and research partners to get to a study design that is workable for both parties. The following conservative assumptions have been made in our power calculations, which were done using the *Optimal Design 1.77 *:

**• α = 0.05 **- Type I error rate of 5%

**• κ = 0.80 **- Power of 80%

**• ρ = 0.05 **- Intra-cluster correlation (ICC) of 5%. This estimate was chosen to be conservative with regards to empirical experience on the most similar previous trial [21], and also with regards to our own baseline data**.

**• n = 60-80 **- see Table 2 for the average cluster size by site

**• J = 15-17 **- see Table 2 for the number of clusters per site

The parameters used implicitly assume that evaluation is undertaken at a household level. For variables measured at the individual level, power is higher due to the increased number of observations per cluster. The resulting standardised MDES were calculated at 0.39-0.40, meaning that our experiment allows picking up an effect of the magnitude of 0.39-0.40 times the standard deviation of the variable of interest (see Table 2).

### Method of Randomisation

The final step in study design is to determine the process which will assign clusters to either the treatment or control group. Random assignment of clusters is the method most likely to ensure there are no systematic differences between the two groups. Prior to randomisation, it was evident that the trials would inevitably take place in an unblinded environment, i.e. both treatment and control group members would be aware of their status (as would researchers and implementers). For any configuration of clusters, villages in the treatment and control clusters are proximate to each other and social, familial, business and political linkages can exist across them. Knowledge of CBHI will inevitably become available to the control population. Unblinded trials can contain risks of bias - of particular concern was the risk that control population members with a strong preference for inclusion in the CBHI program would push scheme administrators to register them, or change their health care seeking behaviour/answers during data collection because of their awareness of the insurance schemes [22]. Moreover, ethical concerns were expressed by implementers about precluding members from access to a potentially beneficial program (this is a common justification for stepped-wedge randomisation [23]). To forestall the risk of bias of results, and assuage ethical concerns, it was decided to assign treatment on a "stepped-wedge" basis [24]. Three waves of treatment will be undertaken, with one third of clusters randomly assigned for treatment in each wave. By the end of the project, all clusters will receive treatment. Prior to wave 1, it was publicly announced that all villages would be included in the scheme, but that implementation would be gradual.

Pre-randomisation matching of clusters was considered, in order to improve balance on important observable characteristics between treated and controls groups. As there are few clusters and many variables we would like to match on, we employed a method for reducing the dimensionality of the matching characteristics, similar to propensity score matching techniques [25]. For each site, a probit regression was run at the household level, with a binary indicator of any healthcare use in the last one month as dependent variable, against a variety of socioeconomic, health and demographic variables^†† ^. Data was taken from the baseline survey (see section 3.4.1). The linear predictions from this probit model were then used to rank clusters: clusters with similar predicted outcome values can be expected to have broadly similar values of the determinants of health care use. The top three ranked clusters were matched into one triplet, the next three into a second triplet and so on. Within each triplet, one cluster is then randomly assigned to the first treatment wave, one to the second and one to the third. In this manner, we ensure that clusters in the three groups are likely to be similar in the matching variable, and therefore also likely to have similar observable characteristics.

Table 3 and Table 4 show means of selected variables for treatment and control groups (for wave 1)^†† ^generated using a simple random draw and the matching procedure described above. As the main outcomes of interest in these studies are related to health care use, supply of health care and health status (see section 4.5), it is especially important to achieve balance on these variables. Generally there are few significant differences in observable characteristics between treatment and control groups across the three sites, with the exception of variables related to health care supply (especially in Pratapgarh), and religion/caste. Matching induces little improvement in balancing as compared to simple randomisation, and given the decrease in power that typically results from using a matching design with limited clusters numbers [26], research staff used a simple random draw (utilizing the sample routine available in Stata 11) to define the order in which clusters were offered CBHI at Kanpur Dehat and Vaishali (see Figure 3, panel 3 for randomisation at Kanpur Dehat).

**Table 3:** Means of selected covariates across treatment and controls (in wave 1)

	Random draw								
	
	Kanpur Dehat			Pratapgarh			Vaishali		
	
	treated	control	p-value	treated	control	p-value	treated	control	p-value
Female (1/0)	0.48	0.49	0.22	0.52	0.53	0.27	0.53	0.52	0.52
Education (years)	4.83	4.95	0.48	4.30	4.30	0.97	3.34	3.08	0.07
Middle wealth third^† ^(1/0)	0.35	0.35	0.90	0.40	0.31	**0.00**	0.27	0.38	**0.00**
Highest wealth third^† ^(1/0)	0.37	0.34	0.33	0.29	0.33	0.10	0.34	0.30	0.22
Scheduled Tribe (1/0)	0.04	0.06	0.08	0.02	0.04	0.11	0.03	0.01	**0.01**
Scheduled Caste(1/0)	0.24	0.19	0.05	0.34	0.34	0.83	0.36	0.28	**0.00**
Other backward caste(1/0)	0.50	0.54	0.22	0.44	0.41	0.37	0.51	0.63	**0.00**
General Caste (1/0)	0.14	0.13	0.78	0.07	0.09	0.17	0.07	0.03	**0.00**
Nomadic Tribe (1/0)	0.09	0.08	0.64	0.13	0.12	0.33	0.03	0.05	**0.04**
age 0-5 years (1/0)	0.11	0.11	0.82	0.12	0.11	0.19	0.15	0.16	0.28
age 5-18 years (1/0)	0.35	0.35	0.74	0.37	0.37	0.97	0.35	0.35	0.55
age 50 years and older (1/0)s	0.14	0.14	0.65	0.16	0.16	0.64	0.13	0.13	0.64
reporting excellent or good health (1/0)	0.60	0.61	0.46	0.59	0.60	0.16	0.48	0.50	0.16
number of acute illness in last 30 days	1.35	1.37	0.85	1.18	1.10	0.21	1.12	1.00	**0.02**
self employed agriculture (1/0)	0.23	0.19	**0.00**	0.07	0.07	0.56	0.08	0.07	0.63
self employed business (1/0)	0.03	0.03	0.80	0.04	0.06	**0.02**	0.06	0.06	0.81
not working (1/0)	0.46	0.49	**0.03**	0.55	0.53	0.07	0.53	0.53	0.88
Salaried worker (1/0)	0.02	0.02	0.52	0.02	0.03	0.14	0.01	0.01	0.54
occasional salary (1/0)	0.05	0.05	0.38	0.11	0.11	0.89	0.12	0.12	0.81
number of chronic illness in last 30 days	0.73	0.84	0.07	1.44	1.31	**0.04**	0.71	0.80	0.09
inpatient facility within 30 min (1/0)	0.15	0.24	**0.00**	0.58	0.55	0.31	0.75	0.75	0.83
outpatient facility within 15 min (1/0)	0.38	0.45	**0.01**	0.52	0.51	0.77	0.56	0.69	**0.00**
pharmacy within 15 min (1/0)	0.36	0.45	**0.00**	0.52	0.51	0.77	0.53	0.63	**0.00**
minimum time to a health facility (minutes)	23.21	20.41	**0.04**	16.82	15.88	0.10	14.58	12.36	**0.00**
not got care when needed (1/0)	0.17	0.16	0.76	0.22	0.23	0.59	0.18	0.19	0.69

A simple random draw is used to draw 3 equally sized groups of clusters.

*^† ^Information on asset ownership and housing characteristics were combined to estimate a wealth index using principal component analysis and divide the population in wealth thirds *[39]

**Table 4:** Means of selected covariates across treatment and controls (in wave 1)

	Random draw with matching								
	
	Kanpur Dehat			Pratapgarh			Vaishali		
	
	treated	control	p-value	treated	control	p-value	treated	control	p-value
female	0.47	0.49	0.06	0.52	0.53	0.37	0.53	0.52	0.62
education year	4.89	4.91	0.86	4.49	4.22	**0.05**	3.58	2.97	**0.00**
middle asset third	0.36	0.34	0.63	0.32	0.35	0.23	0.29	0.37	**0.01**
highest asset third	0.38	0.33	0.09	0.37	0.29	**0.00**	0.39	0.28	**0.00**
Scheduled Tribe (1/0)	0.03	0.07	**0.00**	0.04	0.03	0.67	0.02	0.02	0.70
Scheduled Caste(1/0)	0.21	0.21	0.97	0.43	0.30	**0.00**	0.24	0.33	**0.00**
Other backward caste(1/0)	0.61	0.47	**0.00**	0.37	0.45	**0.00**	0.60	0.59	0.93
General Caste (1/0)	0.08	0.17	**0.00**	0.06	0.09	0.09	0.06	0.03	**0.01**
Nomadic Tribe (1/0)	0.08	0.08	0.78	0.11	0.13	0.40	0.08	0.03	**0.00**
age 0-5 years (1/0)	0.10	0.11	0.44	0.12	0.11	0.92	0.15	0.16	0.15
age 5-18 years (1/0)	0.36	0.34	0.28	0.37	0.36	0.78	0.35	0.35	0.57
age 50 years and older (1/0)s	0.13	0.14	0.50	0.16	0.16	0.54	0.13	0.13	0.91
reporting excellent or good health (1/0)	0.60	0.61	0.61	0.61	0.59	0.37	0.50	0.49	0.31
number of acute illness in last 30 days	1.41	1.33	0.26	1.21	1.10	0.11	1.08	1.02	0.27
self employed agriculture (1/0)	0.23	0.19	**0.00**	0.07	0.07	0.79	0.07	0.08	0.44
self employed business (1/0)	0.03	0.03	0.94	0.06	0.05	**0.05**	0.07	0.06	0.05
not working (1/0)	0.47	0.48	0.49	0.52	0.54	0.15	0.53	0.53	0.84
Salaried worker (1/0)	0.02	0.02	0.51	0.03	0.02	**0.01**	0.01	0.01	0.07
occasional salary (1/0)	0.05	0.06	0.20	0.10	0.11	0.53	0.10	0.12	0.09
number of chronic illness in last 30 days	0.76	0.82	0.30	1.32	1.37	0.40	0.77	0.77	0.96
inpatient facility within 30 min (1/0)	0.18	0.22	0.18	0.53	0.58	0.09	0.76	0.74	0.63
outpatient facility within 15 min (1/0)	0.38	0.45	**0.02**	0.59	0.48	**0.00**	0.62	0.66	0.10
pharmacy within 15 min (1/0)	0.44	0.40	0.22	0.66	0.44	**0.00**	0.60	0.60	0.89
minimum time to a health facility (minutes)	20.39	22.11	0.22	14.13	17.13	**0.00**	13.22	13.00	0.73
not got care when needed (1/0)	0.02	-0.10	0.47	0.21	0.23	0.36	0.19	0.19	0.95
female autonomy index	1772.50	1577.70	0.48	0.25	-0.17	**0.01**	-0.18	-0.01	0.22

This random draw procedure was modified for Pratapgarh, which has a small number of villages (15) and highly varying numbers of SHG members per village. This situation makes it impossible to define clusters of villages as described in section above. Therefore, at Pratapgarh, we created 5 triplets of 3 villages each, by ranking villages according to the number of SHG members within each village, then placing the three largest in one triplet, and so on. In each triplet of villages, one village was then randomly assigned to each of the three treatment waves. This procedure is expected to involve a smaller loss of power than pure matching, due to the smaller number of degrees of freedom taken up [27].

### Outcome Measures & Data Collection

These trials follow a mixed methods design, collecting quantitative, qualitative and spatial data on trial impact. The quantitative outcome indicators being tracked offer a holistic coverage of the key impact areas in CBHI: health services utilisation levels, healthcare expenditure and financing patterns, equity in healthcare use, and households' socio-economic status. Parallel programs of spatial and qualitative research provide complimentary evidence on these areas, and additionally examine impact areas which are not amenable to purely quantitative analysis (e.g. social capital, or physical access to care). This section describes all tools used for data collection. These will be administered in all 3 waves over the course of the study, with each research wave being undertaken immediately prior to a wave of implementation. A longitudinal database is thus built up in each research strand, allowing evaluators to map the evolution of the impact of insurance over time. All tools were drafted with input from a scientific advisory committee and researchers with prior field surveying experience on related topics. Ethical approval for all tools was gained from the independent ethics committee of the University of Cologne. Informed consent was taken prior to each interview, and respondents were free to halt the interview at any time, or to refuse to answer questions within it. All baseline surveying took place prior to the release of information about the CBHI project to the community, such that the baseline data is not biased by knowledge of the experiment.

#### Quantitative data collection

The core quantitative tool is a Household (HH) survey. This survey covers a standard set of demographic variables, along with brief self-reports of health status (drawn from the EQ-5D tool) and a vulnerability module [28]^§§ ^. Core sections record illness-level data on health-seeking behaviour, expenditure levels and financing methods. The survey contains an exceptional amount of detail on health care seeking behavior and financing, as these are the main topics of study, but also because detailed information on frequency of visits and associated costs is needed for the calculation of insurance premia. HH socio-economic status is captured via questions on monthly non-health expenditures and asset ownership, similar to the formats used by the Indian government's National Sample Survey Organisation.

Baseline data collection took place from March to May 2010. The tool was administered to all households in both treatment and control groups, with either the SHG member or the head of household as the primary respondent. A separate, additional sampling frame of non-SHG households from the target villages was constructed at each site, and 1500 non-SHG households selected for interview, via a random draw, using village population to weight interview numbers per village. Research staff remained on site throughout data collection, supervising widespread random spot-checks of completed forms. Completed forms were entered into a relational database via a customised CS-Pro based interface. Electronic data was again randomly re-checked against original physical forms. 3,686 SHG households were interviewed and 1,562 non-SHG households. The overall response rate among SHG members was 87%, as some households contained more than one SHG member, and some were not available at the time of interview. The response rate among non-SHG members was 100%, as unlike SHG members, non-responding households were replaced with an alternate household.

#### Qualitative data collection

A parallel program of qualitative research explores similar key areas. This research stream examines some areas not amenable to other forms of research e.g. social capital. It also serves to identify knowledge gaps to be filled by other methods; to probe and explain contextual differences in relationships identified by quantitative investigation; to provide comparative and/or corroborative evidence for quantitative conclusions and interpretative data in case of failures; and to detect unexpected side-effects. Research is divided into two strands: Health Issues and Social Capital & Risk Management. Health Issues covers understanding of health problems, healthcare seeking behaviour, and patterns of healthcare expenditure. Social Capital & Risk Management includes types of risk experienced, coping mechanisms, healthcare financing mechanisms, insurance knowledge, and community dynamics.

Both sets of topics are examined via semi-structured focus group discussions (FGDs) and key informant interviews (KIIs). FGDs are held with the SHG members and male head of the SHG households. Key-informant interviews are administered individually to healthcare providers, SHG leaders and community leaders. A separate version of each tool is used depending on type/category of respondent. To select respondents, SHG groups are stratified into three categories according to their distance from modern healthcare, and equal numbers of groups are then selected randomly selected from each category, allowing researchers to capture the range of conditions across the study area. Research staff remained on site throughout data collection, providing extensive field supervision and feedback to the field investigators. 104 FGDs and 91 KIIs for health issues were completed across all three sites during baseline. 72 FGDs and 18 KIIs on social capital and risk protection were completed.

#### Spatial data collection

Baseline spatial surveying took place from May-June 2010. At each site, village boundaries and road networks were mapped with the aid of GIS, and locations of all healthcare providers were recorded via handheld Global Positioning System (GPS) recorders. Once mapped, a quantitative health care provider (HCP) questionnaire was administered to the manager or senior medic at each provider. The HCP questionnaire catalogues services provided, facilities available, staffing levels and prices of each provider. The questionnaire was administered to all healthcare providers located in the block (a medium-sized Government of India administrative region) in which the projects are taking place, along with secondary and tertiary providers located in close proximity to the block. All forms of providers are covered, including unqualified providers of modern healthcare, traditional healers and spiritual healers.

All data collection was performed under field supervision from research staff, and all forms back-checked in situ. GPS data was uploaded at regular intervals to track its collection and accuracy. Post-survey, HCP data was double-entered via a customised Microsoft Access format. Detailed GIS maps depicting village boundaries, road networks and provider locations were developed, and HCP data was joined with the GIS maps. 3092 HCP surveys were completed.

#### Process evaluations

On these trials, a long set of activities link the baseline and outcome levels of key impact indicators. A range of associated assumptions as to how the implementation program runs, and the outcome each module generates, underlies the causality asserted by researchers. A "plausibility"[29] or "theory-based"[30] approach, in which the underlying causal chain assumed by the trial is explicitly outlined and empirically tested, is widely suggested as a necessary support to the validity of impact evaluations of such programs. Including such "process" evaluations as an integral part of trial design has been suggested as particularly helpful in multi-site cluster randomised trials of programs, where identical/highly similar interventions may be managed or received differently by different audiences [31]. Therefore, in addition to baseline tools, qualitative and quantitative interventions examining the operation of key elements in the treatment process will be implemented.

## Discussion

### Relationship to other trials

The treatment program on these three trials randomises access to insurance cover; three previous trials which randomise access to micro health insurance have been undertaken, one in Africa [10], one in Latin America [32], and one ongoing in India [33]. However, more fundamentally, these trials apply a program of community action: the treatment population is enjoined in group settings to learn about insurance, before actively making community decisions regarding the content, form, and management of the insurance scheme. The response to treatment is not purely reactive, but interactive. A recent strand of literature has seen CRCTs which randomise participation in structured, interactive programs of participatory community action being developed [34] and conducted [35]. At least one previous study has combined two of these elements (access to a microfinance product, and a participatory community training and action cycle) into a single treatment program [36,37]. It should be noted that while the treatment applied is thematically related to those used in other trials, the overall treatment effect should not be considered as a sum of these parts: the treatment applied in this study is a program, whose effects must be evaluated as a whole [19].

### External validity of trials

CRCTs provide sound quantitative information on the causal effects of an intervention on the outcome measures of interest. However, no randomised trial generates results which are applicable to all persons in all settings [38]. The limits on the external validity and generalisability of findings on these trials are defined by the two stages of selection prior to randomisation: the purposive selection of implementing partners, and the self-selection of local residents into implementing partners' SHG ground structure. Both stages of selection are necessary to establish the conditions required for implementation of this program. It should be made explicit that our experiments will only allow statements on the impact of CBHI on access to care and financial protection within a population of SHGs. Researchers have thus taken steps to ensure that the limits of external validity of the trials can be probed. All quantitative elements of the baseline study were applied to separately selected samples of SHG and non-SHG population in the trial areas, allowing researchers to map in detail observed differences between the two groups. Additionally, the treatment program is replicated in three separate settings, with three almost entirely separate implementation teams. This affords researchers greater confidence in discerning results that are potentially generalisable, by allowing them to search for results which are repeated over trials. Social science RCTs typically run only one trial: in this context, three replications is a considerable advance for external validity.

The only aspect of the institutional environment which has been purposively varied by site is the average length of operation of SHGs. At site 1, most SHGs have been in existence for over a decade. At site 3, most SHGs were specially created for the purposes of the insurance trial, and were in operation for only 9-12 months prior to the beginning of CBHI activities. Trial 2 has average SHG operating lengths of 4-6 years. This variation allows insight into the depth of community involvement necessary to produce effective implementations.

## Conclusion

In this paper, we have described the design of three trials which must randomise access to an insurance scheme within a pre-existing community, but also aim to set up insurance administration structures based on social linkages between treatment group members. We point out several dangers to the validity and accuracy of the measured outcomes of these trials. Of particular concern is that randomisation across community lines can lower treatment uptake, and create conditions that encourage contamination of the control group. These dangers are caused because of the fact that the treatment program must be run as a trial.

In order to avoid these dangers, we have made a unique package of research design decisions. Of particular note is an innovative approach to defining the clusters/units of randomisation used in the trial, which aims to preserve community structures within each cluster as far as possible; this is allied to the infrequently used "step-wedge" randomisation design to dis-incentivise control group members from pushing for inclusion in the scheme.

Two lessons can be drawn, one narrow, and one more general. Narrowly, trials which must subdivide within a community, but seek to preserve community structures, may find that the design described here, or a variant thereof, will serve to increase the robustness of their output. More broadly, this paper can be read as a case study of how the research demands of a randomised trial of a social program have the potential to alter the environment in which the program is implemented. This in turn can alter the impact of the program: so what is measured by the experiment is not what would have occurred if the program had been run non-experimentally. This is a little discussed, but no doubt often encountered issue in the design of randomised trials in the social sciences. Researchers must take care to identify and make their trials robust to these potential feedbacks.

## Competing interests

The authors declare that they have no competing interests.

## Authors' contributions

DD & RR conceived the study, and participated in its design and co-ordination. CD & PP participated in the design of the study and survey tools, and managed data collection. EVDP participated in the design of the study and survey tools. All authors read and approved the final manuscript.

## Endnotes

*Note that in the context of voluntary insurance, self-selection of households into the CBHI scheme still exists. For this reason, it is important to compare those initially assigned to the treatment group to those in the control group, independent of insurance status and measure the intention to treat effect. Longitudinal data collection also offers possibilities to control for selection into the insurance scheme.

^† ^Note that this purposive selection was used to increase the probability of successful uptake of the CBHI schemes, but also means that measures of impact are not necessarily generalisable to the entire Indian SHG population.

^‡^Experimental design took place immediately after baseline surveying was completed

^§^Available from *www.wtgrantfoundation.org/resources/overview/research_tools*.The Cluster Randomised Trials with Repeated Measures module was used, which employs a considerably more complex version of the basic formula presented in the text. Within this more complex formulation we additionally set the parameters F (frequency of surveying) = 1, D (trial duration) = 2. P is not a required parameter.

**ICC was found to be less than 5% for most variables of interest in baseline data (calculated using Stata's loneway routine). This implies lower MDESs than those presented.

^†† ^To avoid having to impose a dependent variable, which obviously affects the weight that is given to each of the variables in creating the matching variable, we also tried using principal component analysis. Results were qualitatively similar, but we prefer using a regression based approach as this has the advantage that the covariates that are highly correlated with the probability to use health care, and therefore important to achieve balance on, get a larger 'weight' in the matching variable.

^‡‡^Note that we define treated as those offered CBHI in the first wave, and controls as those treated at any point after the first wave.

^§§^More information on vulnerability survey scan be found at http://go.worldbank.org/R048B34JF0
